# Case fatality risk of the SARS-CoV-2 variant of concern B.1.1.7 in England, 16 November to 5 February

**DOI:** 10.2807/1560-7917.ES.2021.26.11.2100256

**Published:** 2021-03-18

**Authors:** Daniel J Grint, Kevin Wing, Elizabeth Williamson, Helen I McDonald, Krishnan Bhaskaran, David Evans, Stephen JW Evans, Alex J Walker, George Hickman, Emily Nightingale, Anna Schultze, Christopher T Rentsch, Chris Bates, Jonathan Cockburn, Helen J Curtis, Caroline E Morton, Sebastian Bacon, Simon Davy, Angel YS Wong, Amir Mehrkar, Laurie Tomlinson, Ian J Douglas, Rohini Mathur, Paula Blomquist, Brian MacKenna, Peter Ingelsby, Richard Croker, John Parry, Frank Hester, Sam Harper, Nicholas J DeVito, Will Hulme, John Tazare, Ben Goldacre, Liam Smeeth, Rosalind M Eggo

**Affiliations:** 1 Faculty of Epidemiology and Population Health, London School of Hygiene and Tropical Medicine, London, United Kingdom; 2 The DataLab, Nuffield Department of Primary Care Health Sciences, University of Oxford, Oxford, United Kingdom; 3 Faculty of Public Health and Policy, London School of Hygiene and Tropical Medicine, London, United Kingdom; 4 The Phoenix Partnership (TPP), TPP House, Leeds, United Kingdom; 5 COVID-19 Outbreak Surveillance Team, Public Health England, London, United Kingdom; 6 These authors contributed equally

**Keywords:** SARS-CoV-2, Coronavirus, COVID-19, Variant of concern, Case fatality risk, CFR, Mortality

## Abstract

The SARS-CoV-2 B.1.1.7 variant of concern (VOC) is increasing in prevalence across Europe. Accurate estimation of disease severity associated with this VOC is critical for pandemic planning. We found increased risk of death for VOC compared with non-VOC cases in England (hazard ratio: 1.67; 95% confidence interval: 1.34–2.09; p < 0.0001). Absolute risk of death by 28 days increased with age and comorbidities. This VOC has potential to spread faster with higher mortality than the pandemic to date.

The severe acute respiratory syndrome coronavirus 2 (SARS-CoV-2) variant of concern B.1.1.7 (VOC) was first identified in Kent, United Kingdom (UK) in autumn 2020. Early analysis suggests it is more transmissible than previously circulating forms (non-VOC) [1]. It is now the dominant strain throughout the UK and is increasing in prevalence across Europe [2]. Early reports of increased mortality have not included data on individuals’ comorbidities, and this information is needed to facilitate pandemic planning.

Certain PCR assays for SARS-CoV-2 do not amplify one of the spike protein gene targets in this VOC. Spike gene target failure (SGTF) is therefore a proxy for VOC identification, with greater than 95% sensitivity for VOC diagnosis during the period from 16 November to 11 January [3].

Working on behalf of NHS England, we estimate the risk of death following confirmation of SARS-CoV-2 infection in England, comparing infection with VOC to non-VOC, after accounting for demographic factors and comorbidities. The code and configuration of our analysis is available online (github.com/opensafely/sgtf-cfr-research).

## Study population

Data were drawn from the OpenSAFELY electronic health records secure research platform, covering 40% of England’s population registered with a general practitioner (GP) (see Supplement, part 1). We used linked data from GPs, SARS-CoV-2 testing, vaccination and mortality records (Supplementary Table S1).

We defined as cases those who tested positive for SARS-CoV-2 between 16 November 2020 and 11 January 2021 and followed them until death or 5 February, when follow-up was censored. Vaccinations against SARS-CoV-2 and diagnoses before the study period were exclusion criteria. The SGTF status was known for 184,786 of 441,161 (42%) people with confirmed SARS-CoV-2 infection between 16 November and 11 January (91,775 VOC; 93,011 non-VOC) (Supplementary Table S4). Full details of the design and analysis are available in the protocol (Supplement, part 9). A total of 867 (419 VOC; 448 non-VOC) all-cause deaths occurred before the administrative censor on 5 February 2021. 

The exposure groups were similar demographically (Table 1). The VOC group was younger with a lower proportion of older cases (≥ 80 years: 0.9% in the VOC vs 1.6% in the non-VOC group), with fewer comorbidities (two or more comorbidities: 2.9% vs 3.8%). Non-VOC cases were more frequent in the first 4 weeks of the study period, while VOC cases predominated thereafter. Consequently, median follow-up time was shorter among the VOC group (36 days; interquartile range (IQR): 30–45) than the non-VOC group (57 days; IQR: 40–72).

**Table 1:** Demographic and clinical characteristics of the study population, SARS-CoV-2 B.1.1.7 fatality risk, England, 16 November 2020–5 February (n =184,786)

	Total		Non-VOC cases		VOC cases	
	n	%	n	%	n	%
Total population	184,786		91,775		93,011	
Deaths	867	0.5	448	0.5	419	0.5
Time to death (days)						
Median (IQR)	13.0 (9.0–21.0)		13.0 (8.0–22.0)		14.0 (9.0–21.0)	
Follow-up time						
Median (IQR)	43.0 (33.0–60.0)		57.0 (40.0–72.0)		36.0 (30.0–45.0)	
Epidemiological week of diagnosis						
16 Nov–22 Nov	21,976	11.9	20,854	22.7	1,122	1.2
23 Nov–29 Nov	14,755	8.0	13,432	14.6	1,323	1.4
30 Nov–6 Dec	14,286	7.7	11,576	12.6	2,710	2.9
7 Dec–13 Dec	18,137	9.8	11,703	12.8	6,434	6.9
14 Dec–20 Dec	19,963	10.8	9,043	9.9	10,920	11.7
21 Dec–27 Dec	24,422	13.2	8,246	9.0	16,176	17.4
28 Dec–3 Jan	34,527	18.7	9,477	10.3	25,050	26.9
4 Jan–11 Jan	36,720	19.9	7,444	8.1	29,276	31.5
Sex						
Female	98,099	53.1	49,468	53.9	48,631	52.3
Male	86,687	46.9	42,307	46.1	44,380	47.7
Age group (years)						
0– <18	27,228	14.7	14,310	15.6	12,918	13.9
18– < 30	36,969	20.0	17,302	18.9	19,667	21.1
30– < 40	34,298	18.6	16,782	18.3	17,516	18.8
40– < 50	32,783	17.7	15,904	17.3	16,879	18.1
50– < 60	30,484	16.5	15,261	16.6	15,223	16.4
60– < 70	14,818	8.0	7,587	8.3	7,231	7.8
70– < 80	5,860	3.2	3,116	3.4	2,744	3.0
≥ 80	2,346	1.3	1,513	1.6	833	0.9
Ethnicity						
White	105,428	57.1	52,687	57.4	52,741	56.7
South Asian	21,562	11.7	11,880	12.9	9,682	10.4
Black	4,530	2.5	1,753	1.9	2,777	3.0
Mixed	2,628	1.4	1,175	1.3	1,453	1.6
Other	2,974	1.6	1,351	1.5	1,623	1.7
Missing	47,664	25.8	22,929	25.0	24,735	26.6
Categorical number of comorbidities^a^						
0	158,017	85.5	77,538	84.5	80,479	86.5
1	20,606	11.2	10,768	11.7	9,838	10.6
≥ 2	6,163	3.3	3,469	3.8	2,694	2.9
Index of multiple deprivation quintile						
1 least deprived	36,560	19.8	15,973	17.4	20,587	22.1
2	34,767	18.8	16,000	17.4	18,767	20.2
3	35,181	19.0	16,192	17.6	18,989	20.4
4	38,603	20.9	19,479	21.2	19,124	20.6
5 most deprived	39,675	21.5	24,131	26.3	15,544	16.7

^a^ Comorbidities as defined in Supplementary Table S2.

A full table including all factors adjusted for is given in Supplementary Table S3.

## Relative hazard of death

**Figure 1:**
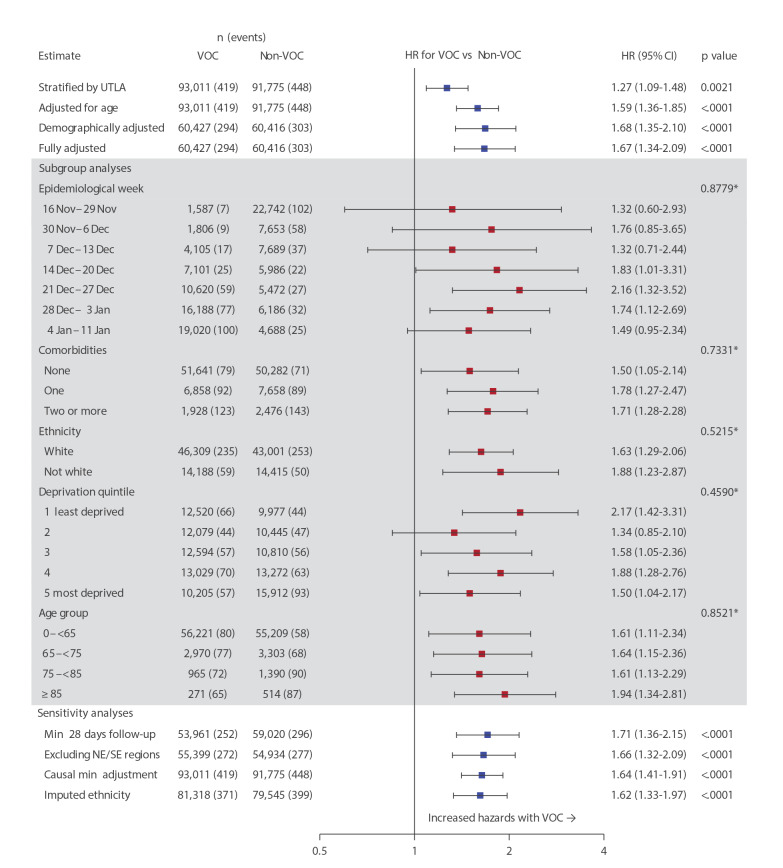
Hazard ratios for death following diagnosis of infection with SARS-CoV-2 VOC vs non-VOC, England, 16 November 2020–5 February (n =184,786)

HR: hazard ratio; IMD: index of multiple deprivation; NE: North East; SE: South East; UTLA: upper tier local authority; VOC: variant of concern.

We calculated the relative hazard of death for VOC compared with non-VOC cases using a Cox proportional hazards regression model stratified by region (upper tier local authority area (UTLA)) [4,5]. Follow-up began at the date of testing positive for SARS-CoV-2 and was censored on 5 February 2021 or 7 days before receipt of a SARS-CoV-2 vaccine, whichever came first. The 7 days prior to vaccination were censored in this analysis to remove a potential immortal time bias because illness which may lead to death would exclude the booking of and administration of a vaccine. Infection with the VOC was consistently associated with an increased hazard of death. In a fully adjusted analysis accounting for demographics and comorbidities, hazards were two-thirds higher in the VOC group (hazard ratio (HR): 1.67; 95% confidence interval (CI): 1.34–2.09; p < 0.0001) compared with non-VOC (Figure 1). Increased hazards for VOC were consistent across all pre-specified subgroup analyses including epidemiological week, age group, categorical number of comorbidities, ethnicity and index of multiple deprivation (IMD) quintile [6]. Increased hazards were also consistent across all pre-specified sensitivity analyses; in an analysis restricted to people testing positive for SARS-CoV-2 infection a minimum of 28 days before the censoring date, the hazard ratio was 1.71 (95% CI: 1.36–2.15; p < 0.0001).

*Likelihood ratio test for interaction between exposure group (VOC, non-VOC) and subgroup.

Cox proportional hazards regression; all models are stratified on region by UTLA; estimating a separate baseline hazard function for each UTLA, with model parameters estimated by maximum likelihood over the full study population.

The demographically adjusted model includes adjustment for: age, sex, IMD, ethnicity, household size, rural urban classification, epidemiological week and care home status. The fully adjusted model includes adjustment for: age, sex, IMD, ethnicity, smoking status, obesity, household size, rural urban classification, comorbidities, epidemiological week and care home status. There was no evidence of non-proportional hazards in this model (global test of Schoenfeld residuals, p = 0.19). The first sensitivity analysis is restricted to people with a minimum of 28 days from testing positive for SARS-CoV-2 to the follow-up censor. The SE and NE NHS England regions are excluded from the second sensitivity analysis (for details see Supplement part 7). The causal minimum adjustment set includes adjustment for: age, care home status, comorbidities, IMD and smoking status.

Missing ethnicity data was imputed for the final listed sensitivity analysis. All subgroup analyses were performed on the fully adjusted model.

## Absolute risk of death by 28 days

**Figure 2:**
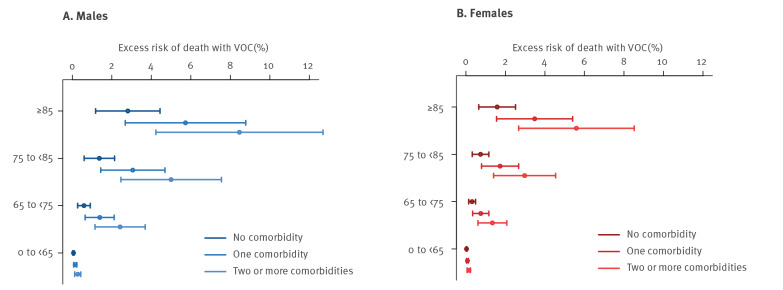
Excess risk of death by 28 days, SARS-CoV-2 VOC compared with non-VOC infection, England, 16 November 2020–5 February (n =112,979)

We found a consistently higher absolute risk of death by 28 days after a SARS-CoV-2-positive test in all groups stratified by age, sex and presence of comorbidities in VOC, compared with non-VOC (Table 2). Risk of death was estimated by the marginal means of a fully adjusted logistic regression model. This analysis was restricted to 112,979 people diagnosed with SARS-CoV-2 a minimum of 28 days before the censoring date, with the outcome death by 28 days after a positive test. Deaths occurring beyond 28 days were censored. Data were not censored 7 days prior to vaccination in this analysis as vaccination is contraindicated in the month following a positive SARS-CoV-2 test. Consistent with the Cox model above, VOC was associated with increased odds of death in this model (adjusted odds ratio (AOR): 1.73; 95% CI: 1.34–2.23; p value < 0.0001, vs non-VOC). The risk of death was low for people younger than 65 years in the absence of comorbidities; in this age group it was higher for male than female cases (VOC: males: 0.14%; females: 0.07% vs non-VOC: males: 0.09%; females: 0.05%). The risk of death was consistently higher for male cases and increased with age and the presence of comorbidities. The highest risk of death within 28 days was seen among those 85 years and older with two or more comorbidities: VOC: males 24.3%; females: 14.7%; non-VOC: males: 16.7%; females: 9.7%). The excess risk of death within 28 days for VOC compared with non-VOC is shown in Figure 2.

**Table 2:** Absolute risk of death by 28 days, SARS-CoV-2 VOC vs non-VOC infection, England, 16 November 2020–5 February (n =112,979)

Sex	Age group (years)	Non-VOC		VOC	
		%	95% CI	%	95% CI
No comorbidities					
Female n = 52,718	0– < 65	0.05	0.03–0.06	0.07	0.06–0.09
	65– < 75	0.45	0.30–0.59	0.72	0.50–0.95
	75– < 85	1.08	0.71–1.45	1.73	1.15–2.31
	≥ 85	2.36	1.47–3.25	3.75	2.34–5.16
Male n = 42,724	0– < 65	0.09	0.07–0.11	0.14	0.11–0.17
	65– < 75	0.85	0.59–1.12	1.37	0.96–1.77
	75– < 85	2.03	1.35–2.71	3.24	2.19–4.30
	≥ 85	4.38	2.72–6.03	6.87	4.33–9.42
One comorbidity					
Female n = 6,858	0– < 65	0.11	0.08–0.15	0.18	0.13–0.24
	65– < 75	1.09	0.78–1.41	1.75	1.25–2.25
	75– < 85	2.60	1.84–3.35	4.13	2.94–5.32
	≥ 85	5.54	3.77–7.31	8.64	5.91–11.38
Male n = 6,661	0– < 65	0.22	0.15–0.28	0.35	0.25–0.45
	65– < 75	2.06	1.51–2.62	3.29	2.44–4.14
	75– < 85	4.81	3.48–6.14	7.54	5.52–9.55
	≥ 85	9.94	6.87–13.01	15.10	10.63–19.58
Two or more comorbidities					
Female n = 1,921	0– < 65	0.21	0.14–0.28	0.34	0.22–0.45
	65– < 75	1.99	1.41–2.57	3.18	2.27–4.09
	75– < 85	4.66	3.45–5.87	7.31	5.42–9.20
	≥ 85	9.65	7.01–12.29	14.68	10.73–18.63
Male n = 2,097	0– < 65	0.40	0.27–0.52	0.64	0.44–0.84
	65– < 75	3.72	2.74–4.69	5.87	4.38–7.35
	75– < 85	8.44	6.44–10.44	12.93	9.99–15.87
	≥ 85	16.65	12.42–20.88	24.34	18.55–30.13

Absolute risk is calculated from the marginal means of a fully adjusted logistic regression model with outcome death by 28 days after positive test for SARS-CoV-2, restricted to the population with a minimum of 28 days from testing to the follow-up censor. Deaths beyond 28 days were censored. The fully adjusted model includes adjustment for: age, sex, index of multiple deprivation, ethnicity, smoking status, obesity, household size, NHS England region, rural/urban classification, comorbidities, epidemiological week and care home status.

VOC: variant of concern.

The figure shows risk difference of death by 28 days for VOC compared with non-VOC, with 95% confidence interval.

## Discussion

The SARS-CoV-2 B.1.1.7 VOC has been the subject of intense research since its emergence. Increased transmissibility means it is now the most common variant in the UK, a trend confirmed here. We found that this VOC was associated with two-thirds higher case fatality than the previously circulating virus in this unvaccinated population. For every three deaths in a population with the previously circulating virus we would expect five deaths in a similar population with VOC. Other studies have assessed the relative mortality of the VOC with similar conclusions [7-10], however, our results are the first to include detailed information on the presence of comorbidities. Interestingly, the effects of age and comorbidities appear to be collinear as adjustment for comorbidities did not alter the findings after adjustment for age. As prevalence of many comorbidities is associated with age, this finding appears plausible [11]. The consistency of the effect for each epidemiological week of diagnosis shows that the increase in mortality due to VOC could not be explained by other secular changes in mortality such as hospitals exceeding capacity.

The absolute risks of death by 28 days demonstrated an increasing risk with age and presence of comorbidities; male cases had a consistently higher risk of death than female cases. However, age and comorbidity risk factors associated with poor non-VOC outcomes appear to be similar to those with this VOC. Therefore, prioritisation for vaccination and shielding can remain the same.

In the UK, all-cause death by 28 days after confirmation of SARS-CoV-2 infection is the standard definition of SARS-CoV-2 mortality [12], so we used death from any cause as the primary outcome. In a sensitivity analysis restricted to people diagnosed with SARS-CoV-2, a minimum of 28 days before the censoring date and logistic regression with deaths censored beyond 28 days, the results were consistent.

This analysis includes people testing positive for SARS-CoV-2. People with asymptomatic or mild infection may not present for testing. Consequently, our estimates of absolute risk of death by 28 days may be overestimates of the true case fatality ratio. In addition, SARS-CoV-2 tests performed in hospital settings in the UK are not tested for PCR S-gene target failure and are therefore not included.

This VOC is now prevalent across Europe and is likely to become the most frequent variant following the pattern seen in the UK [2]. Policymakers and pandemic planners need to account for higher mortality of this VOC.

Crucially, emerging data suggest that the currently approved vaccines for SARS-CoV-2 are effective against the B.1.1.7 VOC [13]. This study highlights the importance of robust national vaccination programmes and infection control measures to contain the SARS-CoV-2 pandemic. Unmitigated spread of the B.1.1.7 VOC has the potential to be both faster and more deadly than the pandemic to date.

## Supplementary Data

1216000 Supplement
